# Malaria Antigen Shedding in the Breast Milk of Mothers From a Region With Endemic Malaria

**DOI:** 10.1001/jamapediatrics.2019.5209

**Published:** 2020-01-06

**Authors:** Lieke W. J. van den Elsen, Valerie Verhasselt, Thomas Egwang

**Affiliations:** 1 The University of Western Australia School of Molecular Sciences, Perth, Australia; 2 inVIVO Global Network, Research Group of the Worldwide Universities Network, Leeds, United Kingdom; 3 Uganda Human Milk and Lactation Center, Med Biotech Laboratories, Kampala, Uganda; 4 Med Biotech Laboratories, Kampala, Uganda

## Abstract

This study examines the association of malaria exposure to malaria antigen in breast milk among lactating women with asymptomatic malaria.

More than 200 million cases of malaria occur yearly, with most in Africa, where infants younger than 5 years account for two-thirds of all malaria deaths.^1^ This highlights the need for successful prevention of malaria infection, especially in early life. Breastfeeding is the most efficient way to prevent child morbidity and mortality attributable to respiratory and gastrointestinal tract infectious diseases.^2^ In contrast, there is conflicting evidence on malaria prevention by breastfeeding.^3,4,5^ Mouse and human data have shown that the presence of foreign antigens in breast milk, such as allergens or viral antigens, could elicit strong immune responses in offspring who are breastfed.^6^ Therefore, we propose what is to our knowledge an original hypothesis: the presence of malaria antigen in breast milk stimulates antimalarial immune defenses and reduces malaria risk in infants who are breastfed. Here, as a critical first step to address this hypothesis, we investigated whether *Plasmodium falciparum* histidine-rich protein 2 (pHRP-2) and lactate dehydrogenase (pLDH) are detectable in the breast milk of mothers from Uganda, a country with endemic malaria.^1^

## Methods

This study included mothers who were lactating and who visited our malaria clinic at St Anne Health Center III, Katakwi District, northeastern Uganda, during the high or low malaria-transmission seasons. Five-milliliter samples of breast milk and fingerprick blood samples were collected after the mothers provided informed consent. Ethical approval for the study was provided by the Uganda National Council for Science and Technology.

The blood samples were used immediately to detect asymptomatic malaria by an ultrasensitive *P falciparum* HRP-2–based rapid diagnostic test (uRDT) (Alere Malaria Ag P.f [Standard Diagnostics Inc]). The presence of malaria antigens in breast milk samples was investigated by *P falciparum*–specific pHRP-2 and pLDH enzyme-linked immunosorbent assays (Quantimal CELISA [Cellabs]), with protocol adaptation (detection levels were 1.2 pg/mL and 4.8 units/mL, respectively).

Data analyses were performed with Prism version 6 (GraphPad Software). We used 2-sided Fisher exact tests to address differences between groups, and *P* values less than .05 were considered significant. Data collection and analysis occurred from March 2018 to December 2018.

## Results

A total of 123 mothers who were lactating visited the malaria clinic during the low malaria-transmission season; an additional 201 visited during the high transmission season. The overall mean [SD] age, body mass index (calculated as weight in kilograms divided by height in meters squared), and lactation duration of the mothers analyzed in this study were 26.2 [6.8] years, 23.6 [2.8], and 12.3 [5.5] months, respectively.

**Figure 1.**
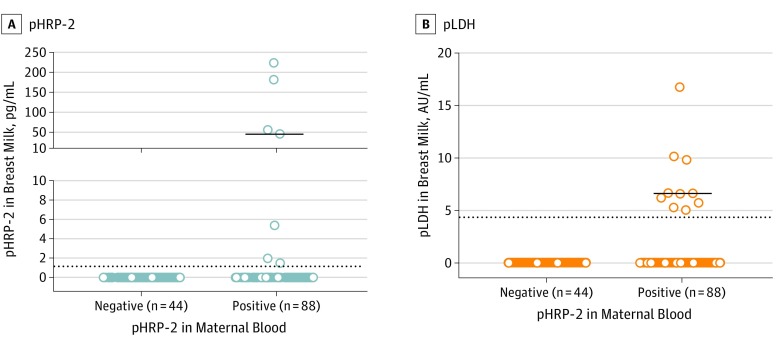
*Plasmodium falciparum* Histidine-Rich Protein 2 and Lactate Dehydrogenase From Plasmodium Falciparum Are Present in Breast Milk From Mothers With Asymptomatic Malaria Data show the concentrations of *Plasmodium falciparum* histidine-rich protein 2 (pHRP-2) and lactate dehydrogenase (pLDH) in breast milk samples from mothers positive vs negative for asymptomatic malaria, as gauged by detection of pLDH in their blood by an ultrasensitive rapid diagnostic test in the absence of malaria clinical symptoms. Dotted lines indicate the limits of detection of pHRP-2 and pLDH antigens in breast milk, as determined by enzyme-linked immunoabsorbent assays. Solid lines indicate the median values among samples with detectable values.

None of the mothers had clinical malaria. When malaria transmission was low and high, 14 of 123 women (11.4%) and 74 of 201 women (36.8%), respectively, harbored asymptomatic malaria (*P* < .001). Among the 88 breast milk samples from mothers with asymptomatic malaria, 7 had detectable pHRP-2 (7.9%) with a median (interquartile range) level of 45.0 (2.0-180.2) pg/mL, and 10 had detectable pLDH (11.3%) with median (interquartile range) values of 6.6 (5.6-9.9) arbitrary units/mL (Figure 1). Overall, 14 breast milk samples (15.9%) were positive for either pLDH or pHRP-2, and 3 (3.4%) were positive for both pLDH and pHRP-2. Forty-four milk samples from mothers without malaria were used as control samples, and none of these showed detectable pHRP-2 or pLDH antigens (Figure 1).

**Figure 2.**
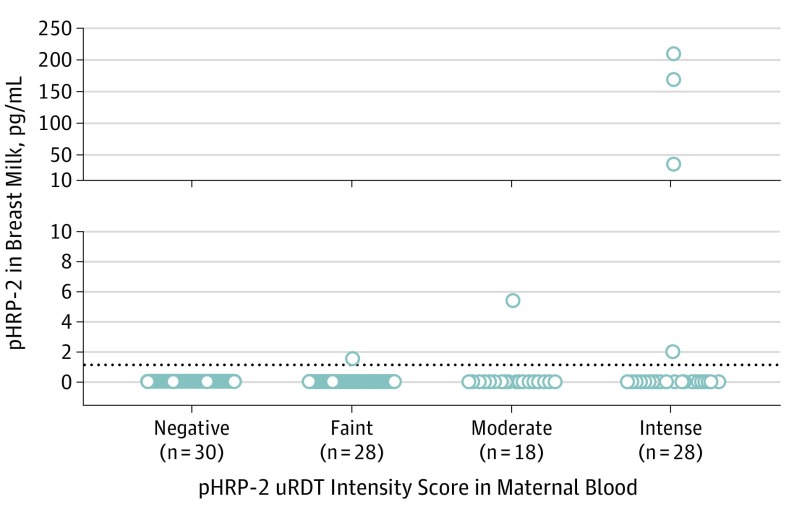
*Plasmodium falciparum* Histidine-Rich Protein 2 Levels in Breast Milk and Association With Levels in Maternal Blood Data show the concentrations of *Plasmodium falciparum* histidine-rich protein 2 (pHRP-2) in breast milk samples from mothers negative for malaria or with various levels of *P falciparum* parasites in their blood, as gauged by the intensity of test bands of the ultrasensitive rapid diagnostic test (uRDT).

To address whether the detection of malaria antigens in breast milk depended on the density of *P falciparum* parasites in mothers’ blood circulation, we categorized the intensity of the test bands of the uRDT readout for 74 malaria-positive blood samples as faint, moderate, or intense, as a proxy measure of parasite density. In the faint category, 1 of 28 samples was positive, with a value of 1.52 pg/mL; in the moderate category, 1 of 18 samples was positive, with a value of 5.4 pg/mL; and in the intense category, 4 of 28 samples were positive, with a median (interquartile range) value of 112.0 (12.6-212.3) pg/mL (Figure 2). Further statistical analysis could not be performed because of the limited size sample. These preliminary data suggest that percentage of breast milk samples positive for pHRP-2 and the concentration of pHRP-2 in breast milk increased with the intensity of test bands.

## Discussion

This study shows (to our knowledge for the first time) that 15% of breast milk samples from mothers with asymptomatic malaria contain malaria antigens. Our preliminary data indicate that blood levels of malaria antigens determine their levels in breast milk. These findings may have important implications for child susceptibility to malaria, since the levels and the nature of malaria antigens in breast milk may strongly influence immune responses to malaria infections in children who are breastfed. Future studies will need to address the immunological outcomes and malaria risk in infants exposed to 1 or multiple malaria antigens through breast milk. This should pave the way for novel and efficient strategies for malaria prevention that are adapted to early childhood.
